# Architecture and Gene Repertoire of the Flexible Genome of the Extreme Acidophile *Acidithiobacillus caldus*

**DOI:** 10.1371/journal.pone.0078237

**Published:** 2013-11-08

**Authors:** Lillian G. Acuña, Juan Pablo Cárdenas, Paulo C. Covarrubias, Juan José Haristoy, Rodrigo Flores, Harold Nuñez, Gonzalo Riadi, Amir Shmaryahu, Jorge Valdés, Mark Dopson, Douglas E. Rawlings, Jillian F. Banfield, David S. Holmes, Raquel Quatrini

**Affiliations:** 1 Fundación Ciencia & Vida, Santiago, Chile; 2 Facultad de Ciencias Biologicas, Universidad Andres Bello, Santiago, Chile; 3 Centro de Bioinformática y Simulación Molecular, Facultad de Ingenieria, Universidad de Talca, Talca, Chile; 4 Center for Systems Biotechnology, Fraunhofer Chile, Santiago, Chile; 5 Centre for Ecology and Evolution in Microbial Model Systems (EEMiS), Linnaeus University, Kalmar, Sweden; 6 Department of Microbiology, University of Stellenbosch, Private Bag X1, Matieland, South Africa; 7 Department of Earth and Planetary Science, University of California, Berkeley, Berkeley, California, United States of America; Universidad Miguel Hernandez, Spain

## Abstract

**Background:**

*Acidithiobacillus caldus* is a sulfur oxidizing extreme acidophile and the only known mesothermophile within the Acidithiobacillales. As such, it is one of the preferred microbes for mineral bioprocessing at moderately high temperatures. In this study, we explore the genomic diversity of *A. caldus* strains using a combination of bioinformatic and experimental techniques, thus contributing first insights into the elucidation of the species pangenome.

**Principal Findings:**

Comparative sequence analysis of *A. caldus* ATCC 51756 and SM-1 indicate that, despite sharing a conserved and highly syntenic genomic core, both strains have unique gene complements encompassing nearly 20% of their respective genomes. The differential gene complement of each strain is distributed between the chromosomal compartment, one megaplasmid and a variable number of smaller plasmids, and is directly associated to a diverse pool of mobile genetic elements (MGE). These include integrative conjugative and mobilizable elements, genomic islands and insertion sequences. Some of the accessory functions associated to these MGEs have been linked previously to the flexible gene pool in microorganisms inhabiting completely different econiches. Yet, others had not been unambiguously mapped to the flexible gene pool prior to this report and clearly reflect strain-specific adaption to local environmental conditions.

**Significance:**

For many years, and because of DNA instability at low pH and recurrent failure to genetically transform acidophilic bacteria, gene transfer in acidic environments was considered negligible. Findings presented herein imply that a more or less conserved pool of actively excising MGEs occurs in the *A. caldus* population and point to a greater frequency of gene exchange in this econiche than previously recognized. Also, the data suggest that these elements endow the species with capacities to withstand the diverse abiotic and biotic stresses of natural environments, in particular those associated with its extreme econiche.

## Introduction

The genus *Acidithiobacillus* consists of a group of obligatory acidophilic, Gram-negative, rod shaped bacteria that derive energy from the oxidation of reduced sulfur compounds to support autotrophic growth [1]. *Acidithiobacillus* species are involved in the bioleaching of metal sulfides, the desulfurization of coal and natural gas and the decontamination of industrial wastes, and for all these reasons are considered a biotechnologically relevant group of bacteria [2]. Significant intrinsic diversity, judged in terms of both genetic and physiological heterogeneity, has been recognized within the *Acidithiobacillus* genus. Several molecular typing studies have classified available strains into lineages [3] and specific assignment of some of these linages has recently been revised [4]–[6].

Due to its ability to oxidize reduced sulfur compounds at moderately high temperatures, *Acidithiobacillus caldus* is the primary sulfur oxidizer in coal piles and spoils and in mineral concentrate reactors operating at temperatures above 40°C [7], [8]. Several aspects of its physiology have been studied in representative strains including sulfur oxidation [9]–[13], central carbon metabolism [14], resistance to arsenic, copper, iron and other heavy metals [15]–[20] and attachment and growth on minerals [21]. Also, a number of broad host range plasmids and a Tn21-like transposon have been characterized for the species [15], [22]–[24]. According to these and other studies strain specific properties are apparent and further support the existence of divergent strain lineages within the *A. caldus* species. Yet, very little is known about the underlying genomic diversity of *A. caldus* and its influence in niche adaptation and strain differentiation.

In the absence of publicly available metagenomic datasets that support extensive populational comparative analyses for these biotechnologically relevant bacteria, lineage specific adaptations need to be addressed using comparative genomic approaches. Here, we report the whole genome sequence comparison of the *A. caldus* type strain (ATCC 51756) with that of *A. caldus* strain SM-1 [14]. The type strain was originally isolated from a coal spoil at the Kingsbury mine in UK after enrichment culture at pH 2.8 [25] and the SM-1 strain was obtained from a reactor used in low grade gold-bearing concentrate bioleaching operating at 40–50°C and pH 1.0–1.5 in China [26].

This work presents the first contribution to the elucidation of the species pangenome and the first comprehensive study of the flexible genome of an *Acidithiobacillus* species. Furthermore, it sheds light into the repertoire of mobile genetic elements (MGEs) found in this econiche. Using molecular approaches, we show the occurrence, distribution and excision capacity of these MGEs in the type strain and other cultivated strains of the species from diverse geographical origins, demonstrating that isolates from different parts of the world are consistently variable at the whole genome level. In addition, we predict the potential function of some of the accessory gene products carried by these MGEs and provide inferences on the ecological significance of these functions in strain linages adaptation.

## Results and Discussion

### Global Strain Comparison

**Figure 1:**
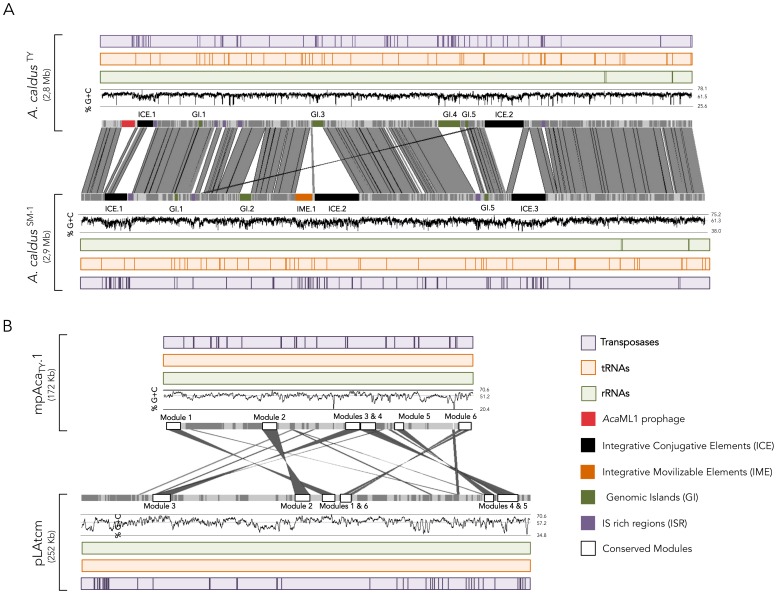
Genomic comparison of *A. caldus* strains ATCC 51756 and SM-1. Similarities and differences between sequenced *A. caldus* strains revealed by *in silico* analysis of (A) the chromosome and (B) the megaplasmids. Chromosomes are represented using ACT. Features are color-coded as follows, grey, CDS or coding sequences; light purple, transposases; light orange tRNAs; light green, rRNA; red, prophage *Aca*ML1; black, ICE or integrative conjugative element; orange, IME or integrative mobilizable element; green, GI or genomic island; purple, ISR or IS rich regions; white, conserved gene modules numbered according to Table S3a.

*A. caldus* ATCC 51756 (*A. caldus* ^TY^) and *A. caldus* SM-1 (*A. caldus* ^SM-1^) are 100% identical at the 16S rRNA gene level and the average nucleotide identity (ANI) of their genomes is 97.9%. The genomes of both strains are compartmentalized in one chromosome, one megaplasmid and a number of smaller plasmids differing in G+C content from the mean value of either chromosome (**Table 1**). The *A. caldus* ^TY^ chromosome is syntenic with that of *A. caldus* ^SM-1^, with similar overall architecture and global structural properties (**Figure 1a**). However, the whole genome comparison shows evidence of several integration events with elements of exogenous origin (**Table 2**). Pairwise BLAST comparisons indicated that the type strain has 633 unique genes while *A. caldus* ^SM-1^ has 872 genes with no homologs in *A.caldus* ^TY^. The majority of the strain-specific genes fall within the chromosomal foreign regions and the additional genome compartments (**Table S1**). One of the foreign elements identified in the type strain's genome is a 72 Kb inducible temperate bacteriophage integrated within *srrA* tmRNA encoding gene whose genomic features have recently been described [27]. The remainder of the foreign elements are described in the sections below.

**Table 1:** Replicons in ATCC 51756 and SM-1 *A. caldus* strains.

	*A. caldus* ATCC 51756				*A. caldus* SM-1				
General Features	Chromosome	mpAca_TY_1	pAca_TY_1	pAca_TY_2	Chromosome	pLAtcm	pLAtc3	pLAtc2	pLAtc1
Accession number	ACVD00000000	ACVD00000000	ACVD00000000	ACVD00000000	NC_015850	NC_015851	NC_015852	NC_015853	NC_015854
Size (Kb)	2,778	171.8	27.5	9.8	2,932	251.8	29.7	14.1	9.8
Total CDS	2,669	215	33	21	2,848	255	10	13	28
Non redundant CDS	2,522	144	33	21	2,635	216	10	13	28
G+C content (%)	61.7	54.4	59.3	50.3	61.0	57.2	59.8	57.7	58.8
rRNA genes	2	0	0	0	2	0	0	0	0
tRNA genes	47	0	0	0	47	0	0	0	0
Prophage regions	1	0	0	0	0	0	0	0	0
Integrated MGEs	6	0	0	0	7	0	0	0	0
Transposases	90	15	2	2	140	50	1	0	1
IS fragments	44	16	1	1	32	7	0	1	1
**Mega/Plasmid Functional Modules**									
Replication/Integration		-	*repA*-like, *kfrA*	*repA*		-	*repA, repC*	*repA, res*	*res1, res2*
Maintenance/Partition		*xerD*	*pasAB, parAB*	toxinX, *copG*, *xerD*		*xerD*	*copG*	*parA, parG*	*vapC, mazE, parA*
Conjugation/Mobilization		*trbN*	*traD, mobA*	-		-	*mobABCDE*	*mobA*	*trbJ, trbL*

Abbreviations: Chr Chromosome; Mpl Megaplasmid; P Plasmid; CDS Protein Coding Sequence; MGEs Mobile genetic elements; IS Insertion sequence. SM-1 plasmid designation is that of You et al., 2011 [14].

**Table 2:** Types of Integrative MGEs in *A. caldus* strains ATCC 51756 and SM-1.

		General features			*Att sites*		MGE Functional Modules			
MGE_ID	Type of MGE	Size (Kb)	# ORFs	G+C %	*attB*	*attL/R*	Phage genes	T4SS	Toxin-Antitoxin System	Partition system
**AcaML1**	Prophage	59,363	72	65.48	srrA	DR (10, 10)	Int, Xis	-		-
**ICEAca_TY_.1**	ICE-1*	71,96	65	56.99	Met-CAT	DR (77, 46)	Int	-	HigB/higA	ParAB
**GIAca_TY_.1**	GI-1¢	13,719	13	58.59	Arg-CCG	DR (77,77)	Int	-	VapC/Phd MazF/MazE	-
**GIAca_TY_.3**	GI-3	45,079	35	58.15	Lys-CTT	DR (76, 45)	Int(t)	-	HipAB VapC/Phd	-
**GIAca_TY_.4**	GI-4	104,967	94	57.00	Ser-GGA, TspO	DR (738, 738)	Int	TraA, TraU		-
**GIAca_TY_.5**	GI- 5&	9,952	11	58,82	Arg-TCT	DR (77, 45)	Int	-		-
**ICEAca_TY_.2**	ICE-2#	183,318	183	57.44	Asn-GTT	DR (77, 47)	Int, Xis, cI, cII, Cro	Trb	VapC/YefM HicA/HicB	ParAB
**ICEAca_SM_.1**	ICE-1*	104,341	98	57.02	Met-CAT	DR (77,46)	Int	Trb	MazF/MazE	ParAB
**GIAca_SM_.1**	GI-1¢	13,644	15	58.53	Arg-CCG	DR (77,77)	Int	-	VapC/Phd ORF/MazE	-
**GIAca_SM_.2**	GI- 2	50,579	45	56.55	Ser-GCT	DR (77, 46)	Int	-	HipAB (2)	-
**IMEAca_SM_.1**	IME-1	80,923	58	56.75	Ser-CGA	DR (89, 18)	Int	VirD2, VirD4, TraY		-
**ICEAca_SM_.2**	ICE- 2#	208,271	202	58.47	Lys-CTT	DR (76,50)	Int, Xis, cI, cII, Cro	Trb	HicA/HicB	ParAB
**GIAca_SM_.5**	GI- 5&	12,358	12	58.42	Arg-TCT	DR (77,45)	Int	-		-
**ICEAca_SM_.3**	ICE-3	157,371	154	56.86	Arg-ACG	DR (77,47)	Int	Trb		PRTRC

(*) ICE_Type 1 elements are >98% similar in about 56 Kb of their sequences;

(#) ICE_Type 2 elements are >90% similar in about 100 Kb of their sequences,

(¢) GI_Type 1 elements are almost identical,

(&) GI_Type 5 elements are almost identical.

Abbreviations: ICE, Integrative Conjugative Element; GI, Genomic Island; IME, Integrative Movilizable Element; Int, Integrase; Int(t), Truncated Integrase; Xis, Excisionase; cI, CI-family phage regulator, cII, CII-family phage regulator; Cro, CRO-family phage regulator; T4SS, Type IV secretion system.

### Origin and Function of Horizontally Transferred Genes in Each Strain

Based on sequence similarity analysis to data in current databases, nearly one third of predicted foreign genes in each strain appears to have originated within the gammaproteobacteria, mostly within the Acidithiobacilli (**Table S2**). Putative horizontally transferred genes with possible origins among members of the betaproteobacteria (49 in strain *A. caldus* ^TY^ and 47 in strain *A. caldus* ^SM-1^) and the alphaproteobacteria (26 and 18 in each strain respectively) were also identified.

**Figure 2:**
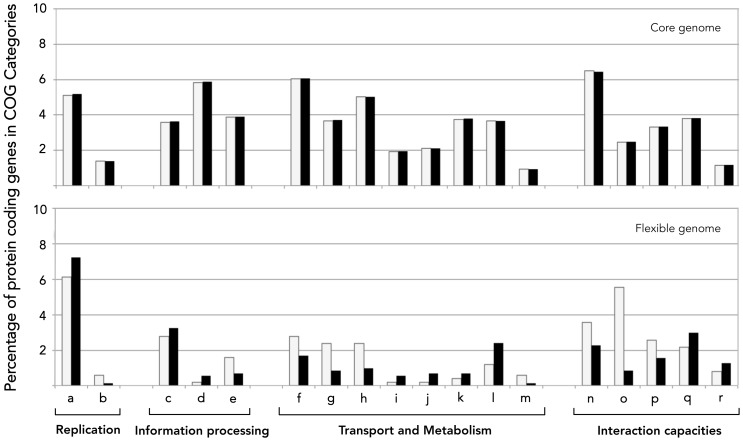
Functional categorization of *A. caldus* strain-specific genes. Bars represent the percentage of gene functions falling into the COG categories indicated. (A) Core genome, (B) Flexible genome of *A. caldus* ATCC 51756 (light grey) and *A. caldus* SM-1 (black). Genes with unknown functions were excluded from both graphs. These represent 35% of the core genome of the species and 64–71% of the flexible genome of *A. caldus* type strain and *A. caldus* SM-1, respectively. (a) Replication, recombination and repair; (b) Cell cycle control, cell division, chromosome partitioning; (c) Transcription; (d) Translation, ribosomal structure and biogenesis; (e) Posttranslational modification, protein turnover, chaperones; (f) Energy production and conversion; (g) Carbohydrate transport and metabolism; (h) Amino acid transport and metabolism; (i) Lipid transport and metabolism; (j) Nucleotide transport and metabolism; (k) Coenzyme transport and metabolism; (l) Inorganic ion transport and metabolism; (m) Secondary metabolites biosynthesis, transport and catabolism; (n) Cell wall/membrane/envelope biogenesis; (o) Cell motility; (p) Signal transduction mechanisms; (q) Intracellular trafficking, secretion, and vesicular transport; (r) Defense mechanisms.

Excluding genes with no predicted functional assignment, which were greatly enriched in *A. caldus* ^TY^ and *A. caldus* ^SM-1^ flexible gene pools, COG gene categories represented in both flexible genomes were enriched in DNA metabolism, cell wall biogenesis, motility, secretion and defense mechanisms (**Figure 2**). In turn, functions related to energy metabolism and inorganic ion transport were differentially represented in the flexible portion of these two genomes (**Figure 2**). Specific functions associated with the flexible gene pool of each strain are described in greater detail bellow.

### Plasmids

The *A. caldus^TY^* plasmid content is distinct form that of *A. caldus* ^SM-1^ both in terms of the number of elements and their gene content (**Table 1**). Unlike all other *Acidithiobacillus* species sequenced so far, both *A. caldus* strains carry a large megaplasmid (>150 Mb). This additional genome compartment has a common backbone made up of 67 highly conserved protein coding genes (97% similar at the predicted amino acid level on average) organized in 6 discrete modules (**Figure 1b**). Within the backbones are genes encoding proteins possibly required for megaplasmid partitioning and replication (ParAB, XerD, GyrAB, ssBP, ssDNA exonuclease, DNA helicase) and several others of unknown function (**Table S3a**). Between 22 and 36% of the megaplasmid-encoded genes have well conserved orthologs in the *A. caldus* chromosome, including nitrogen assimilation and regulation functions, a Kdp potassium transporting ATPase and several transposases (**Table S3a**). This suggests these were recently mobilized from the megaplasmid to the chromosome or vice versa. The larger *A. caldus* ^SM-1^ megaplasmid also carries accessory genes predicted to be involved in iron uptake, heavy metal tolerance, nucleotide metabolism and several pseudogenes.

Typical plasmid replication, maintenance and/or mobilization gene modules were identified on the smaller plasmids (**Table 1** **, Table S3b**). Plasmid pAca_TY_.1 resembles pLAtc2 and pLAtc3 and previously characterized members of the pTcM1 plasmid family [22]. These four plasmids share an 11 Kb region containing genes for replication, partitioning, a Pin-like invertase and an IncQ-like mobilization relaxase of the MobA-type, as well as small highly conserved proteins of unknown function. Plasmid pAca_TY_.2 and pLAtc1 are different from all known Acidithiobacilli plasmids and from each other. Yet, it is likely that pAca_TY_.2 corresponds to pTK1 plasmid reported in the original description of the type strain [28].

### Transposable Elements

**Figure 3:**
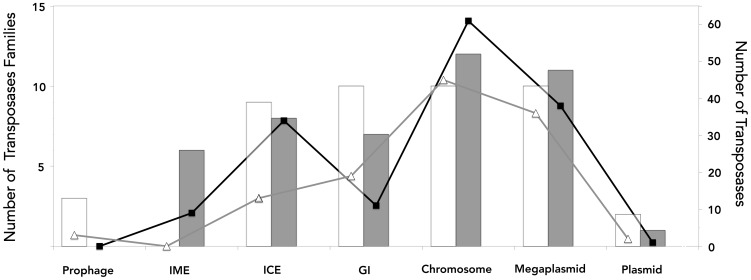
Diversity and abundance of IS families in *A. caldus* sequenced genomes. Bars represent the number of insertion sequence families predicted in the *A. caldus* type strain (white) and SM-1 strain (grey) genomes associated to the core and flexible gene complement. Lines represent the total number of sequences in each category in the *A. caldus* type strain (triangle) and SM-1 strain (square). Abbreviations: ICE, integrative conjugative element; IME, integrative mobilizable element; GI, genomic islands.

Prediction and classification of transposases using TnpPred [29] indicated that nearly 5% of the predicted genes of both strains encode transposases belonging to 20 different IS families (**Table S4a**). Although the diversity of IS types is similar between strains, strain SM-1 has more transposases (total 154) than the type strain (total 97) (**Figure 3**). Four additional IS families were found in the current analysis of strain SM-1 with respect to its genome annotation [14]. The IS family distribution and relative abundance varied between replicons in both strains (**Figure 3**). The most abundant IS families in the chromosome and the megaplasmid were IS5 and IS256 for the type strain and ISL3, IS5 and IS21 in *A. caldus* SM-1.

In both strains, a large number of these IS elements were found within large integrative elements in the chromosome and in the non-shared gene blocks of megaplasmids mpAca_TY_ and pLAtcm. Several of these ISs were found to cluster in flexible genomic regions that do not fit the criteria of genomic islands (GI), integrative conjugative elements (ICE) or even composite transposons. These IS rich regions (ISR) also included accessory functions related mostly to O-antigen biosynthesis, sugar modification and transport (**Table S4b**).

### Integrative Mobile Genetic Elements

Strains *A. caldus* ^TY^ and *A. caldus* ^SM-1^ have 6 and 7 integrated MGEs respectively, 3 of which are fully or partially conserved in both strains (**Figure 1a**). The unique MGEs that differ in genomic integration site, size, G+C, and gene content, have been named according to their position in the core genome of the species. In turn, conserved elements have been similarly named. **Table 2** provides general information on the integrated MGEs detected.

Briefly, all these elements are flanked by pairs of direct repeats (DR, *attL* and *attR*), generally involving tRNA genes and encode a repeat-adjacent integrase (*int*, COG4974), predicted to mediate unidirectional site-specific recombination between the attachment site of the element (*attI*) and the bacterial attachment site (*attB*). Five of these elements encode components of type IV secretion systems (T4SS) of IncP plasmids producing P-type pili (*trb*-type) for conjugative plasmid transfer that define them as ICE (**Table S5**). ICE-2 and ICE-3 type elements have all the essential components of a functional T4SS (VirB1 to VirB11), including the VirD2 relaxase, the VirD4 coupling protein that links the secretion system and relaxosome at the cytoplasmic membrane. One of the MGEs present in the *A. caldus* ^SM-1^ genome encodes only the relaxase and the coupling protein typically present in non-conjugative mobilizable plasmids, and is thus classified as an Integrative Mobilizable Element (IME). The remaining elements flanked by direct repeats identified herein, that are larger than 5 Kb and encode an integrase gene or fragments of it and yet lack clear signatures for mobilization or conjugation, are designated as GIs.

All *A. caldus* ICE elements encode ParAB or ParB/ThiF partitioning protein systems, presumably involved in segregation of the excised forms during host cell division (**Table 2**). Both these system have been demonstrated to play a role in ICE partitioning in other microbes [30], [31]. Also, all predicted ICE elements and some of the GIs harbor addictional modules consisting of a pair of genes encoding a type II toxin-antitoxin system consisting of a stable toxin (VapC, MazF, HigB, HicA, RelE) and a labile antitoxin (YefM, MazE, HigA, HicB, RelB) [32]. Resembling their roles in plasmid maintenance, toxin-antitoxin modules have been suggested to function as ICE post-segregational killing systems [33], [34], yet other roles can not be excluded.

The two largest ICE elements (ICE.2 type) are highly similar to each other over about 50% of their respective sequences (∼100,000 Kb), with at least 90% nucleotide sequence identity. In addition to a well conserved integrase (COG4974), both elements contain an excisionase (COG1257). Also, three ORFs encoding orthologs of the classical cI (COG2932), cII (pfam05269) and Cro (COG4197) phage regulators are conserved between the ICE.2 type elements. The relative levels of these regulators are known to control the lysogenic/lytic switch of phages such as Lambda [35] and ICE such as ICESt1 [36]–[38], suggesting that they may participate in the control of the integration/excision events. However, the integration site and accessory gene complement of both elements is distinct, suggesting that they are members of a family of elements that frequently occur in Acidithiobacilli genomes and confer different functional properties depending on their specific cargo and/or niche (**Table 3**).

**Table 3:** Accessory genes present in *A. caldus* strains ATCC 51756 and SM-1 flexible genome.

Genes/Operon	Description	Function	*A. caldus* ^TY^ MGEs	*A. caldus* ^SM-1^ MGEs
**Ion uptake, homeostasis and/or tolerance**				
*phoO/P*	Phosphate selective porin	Phosphorous assimitation		pLAtc2
*amtB*	Ammonia uptake permease	Nitrogen assimilation	mpAca_TY_1	
*gnlA*	Glutamine synthetase I	Nitrogen assimilation	mpAca_TY_1	
*omr*	TonB-dependent outer membrane receptor	Iron homeostasis		pLAtcm
*mntH*	Mn2+/Fe2+ transporter	Iron homeostasis		pLAtcm
*kdpABCE*	Potassium transporting ATPase	Potassium homeostasis	ICEAca_TY_.1	pLAtcm, ICEAca_SM_.1
*merTPAB*	Mercury resistance	Ion detoxification		IMEAca_SM_.1
*arsADR*	Arsenic resistance	Ion detoxification		pLAtc3
*czcD*	Heavy metals trasport, CDF family protein	Ion detoxification		pLAtcm
*czcCBA*	Heavy metals trasport, RND family efflux pump	Ion detoxification		pLAtcm
*TISS, TolC*	Type I secretion system	Ion detoxification	ISRAca_TY_.6	IMEAca_SM_.1
**Energy metabolism**				
*cyoABCD/ctaAB*	bo3-like terminal oxidase	Growth in low O2 conditions	pAca_TY_1	ICEAca_SM_.1
*ndhDF*	NADH dehydrogenase, subunits 4 & 5	Alternative electron transport		IMEAca_SM_.1
*hupSL-hypFCDEAB*	Group 2 Ni-Fe hydrogenase	Hydrogen metabolism	GIAca_TY_.4	
*hyfBCEFG*	Group 4 hydrogenase	Hydrogen metabolism	GIAca_TY_.4	
*car*	Beta-CA- type Carbonic anhydrase	CO2/pH homeostasis	GIAca_TY_.4	
**Phage/MGEs resistance mechanisms**				
*rms*	Restriction modification system	Defense against MGEs	GIAca_TY_.1, ISRAca_TY_.4	GIAca_SM_.1, ICEAca_SM_.3
*CRISPR/cas*	CRISPR/Cas system	Defense against MGEs	ICEAca_TY_.2	ICEAca_SM_.2
**Substrate colonization**				
*fleQ, flhA-FLEN, flgEFG*	Flagella biosyntehsis	Motility/Adhesion	ICEAca_TY_.2	
*cheYZA, mcp*	Chemotaxis system	Motility/Adhesion	ICEAca_TY_.2	ICEAca_SM_.1
*pilL/NOPQRSUMV*	Type IV adhesion pili	Motility/Adhesion	ICEAca_TY_.2	ICEAca_SM_.2
*wba/wcb*	O-polysaccharide biosynthesis	LPS biosynthesis	GIAca_TY_.3, ISRAca_TY_.1	
*rfb*	Unbranched O-polysaccharide biosynthesis	LPS biosynthesis		GIAca_SM_.2
*gtf*	Gycosyltransferases	LPS/EPS biosynthesis	ISRAca_TY_.2. ISRAca_TY_.3	ISRAca_SM_.2

Abbreviations: ps = pseudogene; CDF = cation diffusion facilitator; RND = resistance-nodulation-cell division.

### Occurrence of Integrated MGEs in *A. caldus* Strains

In order to gain insight into the distribution of the identified ICEs, IME and GIs, their occurrence was analyzed in five strains of *A. caldus* from diverse geographical origins. The presence of the predicted elements was assessed by PCR of the borders (*attL*/*attR*) and/or the integration site in the genome (*attB*) and the nature of the recovered amplicons was confirmed by sequencing (**Table 4**).

**Table 4:** Concurrence and MGEs distribution of *A. caldus* strains.

Strain	AcaML1	ICEAca_TY_.1	ICEAca_TY_.2	ICEAca_SM_.2	ICEAca_SM_.3	IMEAca_SM_.1	GIAca_TY_.1	GIAca_SM_.2	GIAca_TY_.3	GIAca_TY_.4	GIAca_TY_.5
*TY*	+	+	+	−	−, s	−, s	+	−, s	+	+	+
*BC13*	+	+	+	−, s	+	−, s	+	−, s	+	+	+
*F*	+	+	+	+	−, s	+	+	−, s	+	+	+
*CSH12*	+	+	+	−, s	+	−, s	+	−, s	+	−, s	+
*MNG*	−	−, s	−, s	+	+	−, s	+	−, s	−, s	−, s	−, s
*#6*	−	−, s	−, s	+	+	+	+	−, s	−, s	−, s	−, s

(+) *attR* and *attL* present; (−) *attR* and *attL* absent; (s) *attB* present.

Patterns of occurrence of the integrated MGEs do not follow geographic distribution of the strains, but rather their relatedness, as assessed by a resolutive phylogenetic gene marker *era* (unpublished data). Elements that are present in the type strain genome also tend to occur in its closely related strain BC13, and also often in the Australian strain CSH-12 and strain F from South Africa. This is also true in the case of the prophage. In contrast, elements that occur in the Chinese strain SM-1 are recurrently present in strain #6 from South Africa and absent from the rest of the genomes analyzed. The IME occurs more stochastically, being found in the SM-1 strain and two South African strains (F and # 6). MGEs distribution profiles could reflect patterns of occurrence of cognate receptors for temperate bacteriophages such as *Aca*ML1, or mating pair formation determinants in each linage. Also, occurrence could reflect phage related selection patterns. One exception seems to be the genomic island GI-1 which is invariably present in all the strains evaluated. In this case, the most conservative explanation is that its integration probably occurred in a common ancestor and that after gene loss and selective decay, it was subsequently fixed.

### Excision/Integration Capacities of *Acidithiobacillus* ICE/IMEs

**Figure 4:**
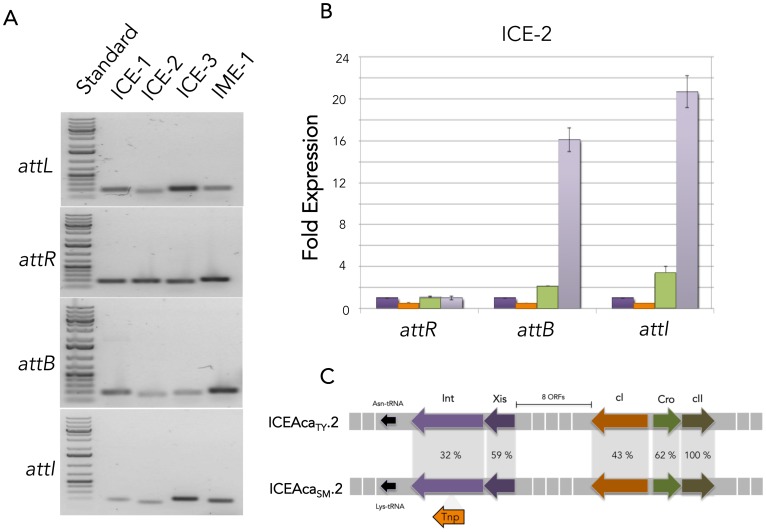
Excision capacities of *Acidithiobacillus* ICE and IME. **A** Agarose gel electrophoresis of end point PCR products generated with primers for *attL*, *attR*, *attB* and *attI* showing excisive recombination of ICE-1, ICE-2, ICE-3 and IME-1 in cognate *A. caldus* strains ATCC51756, F, CSH12 and F respectively (**A**). DNA damaging agents effect on ICE-2 excision frequency (**B**). Relative fold induction of the integrated form (*attR*) and the excised form (*attB, attI*) of ICE-2 in the type strain assessed by real time PCR upon treatment with Mit C or mitomycin C (1 µg/ml, 16 hs), light purple; UV-C radiation (200 J/m^2^, 5 min), light green; and Fe^3+^ or ferric iron (250 mM, 45 min), orange, with respect to control conditions (no DNA damaging treatment), dark purple. Abbreviations: ICE, integrative conjugative element; IME, integrative mobilizable element.

To evaluate the capacity of the detected ICEs and IMEs to excise out of their host chromosomes, end point PCR analysis of genomic DNA obtained from stationary phase *A. caldus* strains was performed. These analyses revealed the co-existence of the integrated (*attL, attR*) and excised (*attB, attI*) forms of all elements in the strains tested (**Figure 4a**). Sequencing of the resulting bands confirmed the nature of the amplicons as that expected from the excision of the cognate elements from their target tRNAs. These results indicate that the elements are capable of excision and formation of a circular intermediate suitable for further transfer to recipient hosts.

Quantitative PCR analysis of the relative ratios of the integrated (*attR*) and excised (*attB*) forms of the ICE-2 type elements (which carry a predicted phage-type regulatory gene module) under control conditions and upon infliction of DNA damage revealed changes in the observed proportions of *attB*/*attI* (**Figure 4b**). This suggests that a phage-type of mechanism underlies ICEAca_TY_.2 and ICEAca_SM_.2 site-specific excision. cI orthologs, like the one encoded in ICE-2 type elements have been found in other ICE, including ICESt1-3 from *Streptococcus thermophilus* [36], ICEBs1 from *Bacillus subtilis* [37], SXT from *V. cholerae* [38] and ICE*Afe*1 from *A. ferrooxidans* [33]. Furthermore, conditions that elicit the SOS (DNA damage) response have been found to derepress the excision of these ICE [33], [36]–[38] and to promote the transfer of ICEBs1 and SXT-related elements from enterobacteria [36]–[39]. Such regulation is reminiscent of DNA damage induced derepression of the site-specific excision of numerous prophages during lytic cycle [40] and suggests that this mechanism also controls the dynamics of integration/excision of ICE-2 type elements in *A. caldus* and possibly also their transfer to a healthy host.

### Functional Clues Derived from *A. caldus* Flexible Genome Analysis

**Figure 5:**
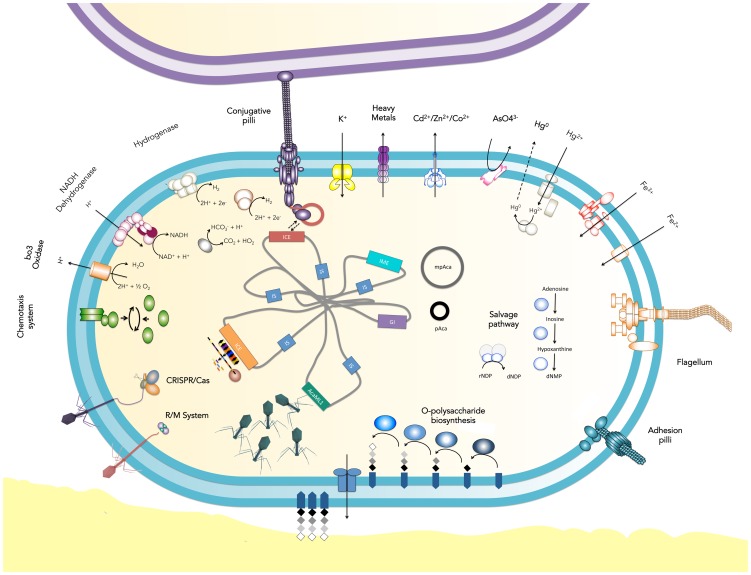
Functional repertoire of the *A. caldus* mobilome. Schematic representation of the predicted shared (full) and exclusive (empty) accessory gene products present in the *A. caldus* ^TY^ and *A. caldus* ^SM-1^ mobilome and the diversity of MGEs identified in this study.

Although each of the *A. caldus* sequenced strains carries a distinct repertoire of MGEs, and despite differences in their origin and culturing histories, accessory genes present in their flexible genomes shared common themes. These genes partitioned in the five mayor functional categories described in detail below. Several of these functional categories have previously been shown to be over-represented in GIs and metagenomic GIs in other model systems and econiches and are related to surface exposed proteins or cellular structures known to be bacteriophage recognition targets [41]. As such many of these functions are probably relevant to population genomics control mediated by bacteriophages prevalent in acidic econiches [42]. In turn, several others reflect differences in niche explotation. MGE-specific distribution of the accessory genes is provided in **Table 3** and gene functions partition between strains is schematized in **Figure 5**.

#### 1. Ion uptake, homeostasis and/or tolerance

Several uptake systems for ions such as ammonium, iron and potassium were found to form part of the *A. caldus* flexible gene pool. Ammonia is a growth-limiting factor in biomining environments. Several copies of the AmtB transporter encoding gene are frequently found in the genomes of bioleaching acidophiles [43], [44] and other free-living bacteria [45]. Although nitrogen fixation genes are commonly associated with MGEs [46], ammonia transport functions are typically chromosomal. The pLAtcm megaplasmid encoded *amtB* may contribute to increased ammonia uptake in acidic conditions.

Conversely, iron loads in acidic econiches can be 10^18^ times the concentration found in neutral pH environments and thus acidophiles like *A. caldus* have to cope with issues related to potential iron toxicity [18]. Encoding additional transporters for ferrous/ferric iron uptake, which tightly regulate metal entrance, may contribute to iron homeostasis. Also, TonB-dependent outer membrane receptors may serve as receptors for colicins and bacteriophages [47], [48] afecting survival and competition in the environment and ultimately genomic stability.

In addition to the potassium transporting ATPase encoding operon *kdpABCF* present in the *A. caldus* core genome, three copies also occur in the flexible genome within ICE.1 type elements and the pLAtcm megaplasmid. The KdpABCF complex is involved in high affinity ATP driven potassium uptake against steep concentration gradients and plays several physiological roles [49]. Increased operon copy number in the Acidithiobacilli may have a primary role in pH homeostasis where an inside positive membrane potential is suggested to be generated by potassium ions [50].

The *A. caldus* ^SM-1^ flexible gene pool was enriched in metal detoxification systems for mercury, arsenic and other less well defined heavy metals. This is probably related to the fact that the SM-1 strain was isolated from gold bearing mineral concentrates [26], which often contain arsenopyrite and other heavy metal entrapped minerals. A truncated version of the mercuric reductase encoding operon *merTPAB* (lacking the regulatory genes *merR* and *merD*) is present in the IMEAca_SM_.1. As shown in other bacteria, unregulated expression of this operon could confer *A. caldus* with increased mercurial detoxification capacities [51]. It has been well established in other microorganisms that horizontal dissemination of genes responsible for resistance to inorganic mercury can occur via MGEs such as transposons, plasmids and GIs in neutrophiles [52]–[54] but no evidence has been reported on their occurrence on IME or ICE type elements nor other MGEs in acidophiles. Conversely, arsenic resistance has been previously described in *A. caldus* and specifically linked to transposon TnAtcArs [15] and plasmid pLAtc3 [14]. In addition, two type I secretion systems (TolC-type) for efflux of antibiotics, dyes or heavy metals, a CzcD cation diffusion facilitator family member and a CzcCBA resistance-nodulation-cell division family efflux pump of Cd^2+^, Zn^2+^ and Co^2+^, also form part of the *A. caldus* flexible genome (**Table 3**). Similar metal detoxification pumps have also been found to form part of the flexible genomes of other free-living bacteria [54], [55]. These findings are in agreement with previous evidence generated for closely related acidophiles like *A. ferrooxidans*, showing that changes in genome structure and gene copy number develop in response to environmental effects and/or toxic metals exposure [56], [57]. In the case of biomining acidophiles, transfer and spread of these functions could contribute to rapid adaptation and survival of the species in highly acidic and metal rich environments containing millimolar quantities of metals [58].

#### 2. Energy metabolism

The flexible gene pool of both strains also contained components of several alternative electron-transporting pathways (**Table 3**). Protein subunits of a terminal oxidase complex encoded in the type strain plasmid pAca_TY_1 are 99% identical to those present in the SM-1 strain ICEAca_SM_.1 and 51–63% identical to the *bo3* quinol oxidase encoded within the *A. caldus* core genome. In addition to the oxidase subunits, the flexible genome gene clusters also encode two heme maturation proteins which convert heme B (protoheme IX) to heme O (CtaA) and heme O to heme A (CtaB) [59], suggesting that this could be a mixed oxidase of the *ba3* type. A *ba3* type oxidase has been identified in the extremely thermophilic bacterium *Thermus thermophilus* HB8 [60] and shown to be expressed under limited O_2_ supply [61]. Since oxygen solubility in water diminishes with temperature (∼25% less at 40°C than at 25°C), this alternative oxidase may have been selected for by *A. caldus* more than once to provide a growth advantage at its higher optimum growth temperature. Alternatively, heme A could replace heme O in the active site of the oxidase depending on growth conditions, as documented in other microorganisms [62], providing additional metabolic flexibility.

The *ndhFD* gene pair encoding essential membrane components of respiratory complex I (NDH-1) occurred in three locations of the *A. caldus* core genome as well as the SM-1 flexible genome. Multiple and divergent copies of *ndhD* and *ndhF* have been reported in other genomes and are suggested to make up many distinct NDH-1 complexes that may feed electrons into the respiratory chain or other electron transport systems conferring further metabolic flexibility [63].

Accessory genes in this functional category also include a group 2 cytoplasmic uptake NiFe-hydrogenase (*hup* operon) and a group 4 hydrogenase (*hyf* operon). Orthodox respiratory *hupSL*-encoded hydrogenase activity is presumed to be responsible for recycling endogenous hydrogen (H_2_) produced during nitrogen (N_2_) fixation in aerobic diazotrophes [64]. Co-occurrence of the *hup* operon with *nifA* and two orthologs of the regulator PII, as well as sequence similarity, suggests that the gene cluster found in GIAca_TY_.4 could have been acquired from a diazotroph. However, lack of N_2_ fixation genes in *A. caldus* type strain [43] suggests that the presumed role of the Ni-Fe hydrogenase is that of a respiratory hydrogenase adapted for use of exogenous H_2_ [65]. The role of the distinct HyfBCEFG hydrogenase present in GIAca_TY_.4 is less obvious. In *Escherichia coli*, the group-4 hydrogenase (hydrogenase-4), is coupled to the fermentative formate dehydrogenase, oxidizing formate to CO_2_ and reducing 2H^+^ to H_2_ under fermentative conditions [66]. This is very likely the case of the hydrogenase-4 HyfBCEFG present in the core genome of both *A. caldus* strains, which co-occur with genes for carbon fixation and presumably interact with the formate dehydrogenase encoded a few genes upstream. However, the flexible genome *hyf* gene cluster is found immediately adjacent to a beta-CA-type carbonic anhydrase encoding gene, the product of which catalyzes the reversible hydration of CO_2_ to bicarbonate (CO_2_+H_2_O = HCO_3_^−^+H^+^) [67]. This suggests that this H_2_-evolving hydrogenase could reduce H^+^ -ions to yield H_2_ (gas) and contribute to pH and CO_2_ homeostasis. Occurrence of alternative terminal oxidases, NDH1 subunits and hydrogenases on MGEs has previously been reported in other microorganisms [54], [68], [69], further indicating that certain adaptive strategies are shared between acidic and neutral environments.

#### 3. Phage/MGEs resistance mechanisms

The two *A. caldus* strains examined carry CRISPR/Cas systems and restriction modification complexes in their flexible genomes. Both features help to build a defense against invading mobile genetic elements and potentially undesirable genetic cargo.

CRISPR loci present in *A. caldus* ^TY^ and *A. caldus* ^SM-1^ ICE.2 type elements have a similar organization, consisting of two repeat–arrays of variable size and spacer content and five genes encoding highly similar Cas-like proteins. In spite of the inter-strain conservation, these proteins are highly divergent from other known and characterized CRISPR associated proteins, including those from its close relative *A. ferrooxidans* [33]. The total number of repeats (23 and 27 respectively) and the diversification of spacer sequences suggests that these loci may be active. None of the spacer sequences is shared between the two loci, yet two spacers from the ICEAca_TY_.2 CRISPR and two others from the ICEAca_SM_.2 CRISPR share sequence similarity to *A. caldus* pTcM-1 family plasmids and one spacer of each locus shares sequence similarity to *Acidiphilium* spp. plasmids with whom the Acidithiobacilli share the niche.

Diverse lines of evidence suggest that CRISPR/Cas cassettes are actively propagated between genomes by horizontal gene transfer. Anomalous nucleotide frequencies of CRISPR/Cas systems compared to cognate chromosomes, conservation of certain *cas* gene arrays in phylogenetically distant genomes and the occurrence of diversified CRISPR/Cas systems in closely related strains or species, are evidence that these cassettes disseminate via horizontal gene transfer as discrete units [70], [71]. Plasmid [72] and GI mediated mobilization [54], [73]–[75] and chromosomal conjugation [75] have been proposed to underlie the dissemination of these cassettes. However, the extent to which these mechanisms operate and actually contribute to intra-species and inter-species CRISPR/Cas propagation remains obscure. The presence of CRISPR/Cas systems within ICE, as described herein for *A. caldus* type strain and strain SM-1 and shown recently also in the case of *A. ferrooxidans* [33], suggests that additional pathways for propagation of chromosomally encoded CRISPR/Cas cassettes exists.

Accessory genes in the *A. caldus* flexible genome (ICE*Aca*_SM_.3 and shared GI.1) also include a type II restriction modification (RM) system, most likely selected for as protection against invading foreign DNA after sequence-specific methylation of defined target sequences of the host chromosome [76]. Type II RM systems occur frequently on mobile genetic elements [77]. The presence and functionality of RM systems such as these may have implications in phage infection and restriction, DNA uptake and natural competence.

#### 4. Substrate colonization

Several cellular functions that play relevant roles in chemo-sensing, surface attachment, autoaggregation and biofilm formation form part of the *A. caldus* flexible genome (**Table 3**) and many other free-living and pathogenic bacteria [78].

In addition to gene clusters in the conserved core genome encoding flagella and chemotaxis genes [14], [43], the *A. caldus* type strain flexible genome carries a 38-gene cluster encoding all necessary genes for basal body, hook and flagella filament biosynthesis [79] and all essential chemotaxis genes that mediate signal specific induced motility [80]. The major structural component of the flagellum, the FlaB flagelin, is less than 70% similar to the core encoded flagelin, suggesting that variable extracellular structures are determined by the presence of this ICE. This probably influences surface attachment [81] or phage recognition [82]. In addition, a 12 ORFs locus encoding type IV adhesion pili, promoting bacterial attachment to, and colonization of a wide variety of surfaces [83] is exclusively present within the ICE.2 type elements.

Three different loci encoding regulation, synthesis and export functions related to the biosynthesis of O-polysaccharide, the outermost domain of lipopolysaccharide (LPS), also form part of the *A. caldus* flexible genome (GIAca_SM_.2, GIAca_TY_.3, ISRAca_TY_.1). In most Gram-negative bacteria, LPS is one of the major constituents of the outer leaflet of the outer membrane. Several bacteria specifically use LPS to adhere to animal and plant host tissues and various abiotic surfaces and recent reports indicate that it has a role in autoaggregation and biofilm formation [84]–[86]. It also serves as a bacteriophage receptor in certain bacteria [87].

Occurrence of all these traits, contributing to different developmental stages of the formation of biofilms, enhance bacterial survival in the environment and provide resistance against a variety of abiotic and biotic stress factors (e.g. bacteriophages). Also, close proximity of the bacterial cells within biofilms has been shown to increase plasmid dispersal by conjugation, as well as DNA release and transformation [88], [89]. No information is yet available on the effect of biofilms in ICEs and IMEs dispersal but it can be expected to be similar to that of conjugative and mobilizable plasmids. In addition, these capacities may improve substrate utilization and thereafter growth and proliferation of *A. caldus* in biomining environments.

#### 5. Nucleotide metabolism

The *A. caldus* ^SM-1^ flexible genome also encodes several functions related to nucleotide salvage to recover bases and nucleosides that result from RNA and DNA degradation.

Specifically, pLAtcm megaplasmid encodes a guanine/adenosine deaminase required for irreversible deamination of adenosine to the nucleoside inosine, a purine nucleoside phosphorylase for deribosylation of inosine to hypoxanthine and a phosphorybosiltransferase which adds activated ribose-5-phosphate to bases creating nucleotide monophosphates. Accessory genes present in ICEAca_SM_.3 also encode the beta subunit of the class Ia aerobic ribonucleotide reductase (*nrdB*). The latter is the small subunit of a functionally critical enzyme complex in nucleotide metabolism that catalyzes the rate limiting step in the synthesis of deoxyribonucleotides used for DNA replication and repair [90]. No reports on the occurrence of ribonucleotide reductases on MGEs other than bacteriophages could be found in the literature [91]. It is possible that an ICE-encoded NrdB subunit (which is well conserved with respect to the chromosomal copy of *nrdB* form *A. caldus* type strain) could activate the chromosomally encoded catalytic subunit NrdA and thereafter supply dNTPs during ICE replication or DNA damage.

## Conclusions

Comparative genomic analysis of *A. caldus* sequenced strains revealed that they differ in about 20% of their respective genomes, which is close to that observed for highly flexible species like *E. coli* [92]. Both strains vary not only in the number and types of independent replicons that they host, but also in the MGE content and accessory gene distribution of their flexible genomes.

In this work we uncovered a considerable diverse repertoire of MGEs, including at least three families of ICE elements, a family of IME elements and several GIs, complementing previous efforts to characterize plasmids [28], insertion sequences [93], and bacteriophages [22] from biomining Acidithiobacilli. Most ICE and the IME are widespread between strains of the species obtained from diverse geographical origins, indicating that conserved or variant versions of them still occur in the *A. caldus* population as active excising elements that may be transferred horizontally when needed. In addition, *A. caldus* SM-1 and type strain are the first Acidithiobailli to be characterized carrying a megaplasmid.

Deep bioinformatic analyses of strain-specific segments allowed identification and functional categorization of the accessory gene pool. Several of the features found (e.g. restriction modification systems, metal resistance systems, hydrogenases, etc.) have been linked to the flexible gene pool in other microorganisms inhabiting completely different econiches, yet facing similar challenges (e.g. bacteriophage infection, limiting nutrients or energy supply) [54], [94], [95]. Yet others, (e.g. mercurial detoxification system, CRISPR/Cas systems) had not been unambiguously mapped to integrative MGEs prior to this report.

Despite differences in the isolation and culturing histories of the two strains under comparison, the nature of several relevant physiological features identified along their flexible genome segments were highly similar (e.g. defense mechanism against MGEs, chemotaxis gene cluster, adhesion pilli gene cluster). However, genes that were common to both flexible genomes were distributed in different types of elements in each strain, indicating that certain features have been selected for more than once in the evolution of the species. Conversely, other functions in the flexible genome clearly reflect the local environmental conditions in which each strain thrives (e.g. several heavy metal homeostasis strategies in strain SM-1).

Collectively, the differences we report here suggest that significant, yet uncharacterized, diversity exists within this particular group of acidophiles and that gain and/or loss of mobile genetic elements and their cargo also plays an important role in strain differentiation and adaptation to acidic econiches.

## Materials and Methods

### Nucleotide sequences

The *Acidithiobacillus caldus* ATCC 51756 draft genome sequence (ACVD00000000, [43]) was assembled using the Phred/Phrap/Consed software package [96]. A total of 41,813 high-quality reads with an average length of 745 bp were assembled into 57 contigs with at least twofold coverage. Remaining gaps were closed by further sequencing gap-spanning PCR products. Final assembly, consisting of one chromosome, one megaplasmid and two smaller plasmids, was deposited at GenBank under the accession number PRJNA36585. Sequences for *A. caldus* SM-1 (NC_015850-54) were obtained directly from the GenBank database.

### Sequence annotation

Gene prediction was performed using CRITICA [97] and Glimmer [98]. Non-coding genes were predicted using tRNAscan-SE [99]. Predicted proteins were functionally annotated using protein alignments to NCBI nr [100] and categorized against the COG database [101] and the GO database [102]. Coding sequences were further characterized using the suite of protein comparison and classification programs available in InterproScan [103] and the Prosite database [104]. Insertion sequence family assignments were made using TnpPred [29]. CRISPRs were predicted using CRISPI [105], CRISPR Finder [106] and CRT1 CRISPR Recognition Tool version 1.1 [107]. Comparative genomic tools such as Microbesonline [108] and RAST [109] were also employed.

### Sequence comparisons

Sequence comparisons were conducted using MUMmer [110], MAUVE [111] and the Artemis Comparison Tool with a minimum score cut-off of 121 and a minimum percentage identity cut-off of 100% [112]. To retrieve paralogs and/or redundant proteins, the predicted proteome of each strain was analyzed against itself using BLASTp and a 95% identity (E<1e-10) as cutoff. To retrieve orthologs predicted proteomes of each strain were cross-compared using bidirectional BLASTp, a reciprocal 95% identity (E<1e-10) cutoff and in-house bioperl scripts. Chromosomal maps were made using the CGview server [113].

### MGE identification

Mobile genetic elements present in the genome of *A. caldus* genomes were identified using an in-house designed pipeline. First, all integrases in the genome of each strain were mapped using BLASTp and a collection of query sequences retrieved from the NCBI database. Second, regions adjacent to each predicted integrase were recovered and their occurrence elsewhere in the genome (as direct repeats) assessed using BLASTn. Regions flanked by the identified repeats were recovered and analyzed for their GC content and skew [114] and occurrence on the other sequenced strain of the species using sequence comparison tools. Finally, gene content of the predicted elements was analyzed to search for hallmarks of horizontal gene transfer.

### Bacterial strains and culture conditions

The bacterial strains used in this study are listed in **Table S6**. *A. caldus* strains were grown in mineral salts medium (MSM) with trace elements [8] and 5 g/L S0 or 5 mM tetrathionate as energy substrate. Cultures were grown at 40°C and pH 2.5 in aerobic conditions (150 rpm). Stock solutions of tetrathionate were filter-sterilized and added to the autoclaved (121°C for 15 min) MSM, whereas ethanol-sterilized powdered sulfur was added to MSM prior to autoclaving at 105°C for 30 min.

### General DNA techniques

*A. caldus* stationary phase cultures to be used for nucleic acid purification, were centrifuged at 6,000 g to remove solid sulfur precipitates prior to cell harvest. The cell pellet was resuspended in 9K salt solution for further washing. Washed cells were collected by centrifugation at 10,000 g for 15 min. DNA isolation and routine manipulations were carried out following standard protocols [115]. DNA quantifications were performed in the NanoDrop 2000 Spectrophotometer (Thermo Scientific).

### End point PCR

Oligonucleotide primers used in this study are listed in **Table S7**. Polymerase chain reaction (PCR) products were amplified with DNA polymerase Dreamtaq (ThermoScientific) and were purified from agarose gels with the Mini Elute Gel Extraction Kit (Qiagen). Each PCR reaction contained 10 ng of template DNA, 0.5 µM of required primers, and 0.2 mM of each deoxyribonucleotide in a volume of 25 µl of 1× PCR buffer containing 1.5 mM MgCl_2_. PCR conditions were as follows: initial denaturing step at 95°C for 2 min followed by 28–30 amplification cycles (denaturation at 95°C for 20 sec, annealing at the appropriate temperature depending on the specific primers pairs for 20 sec and elongation at 72°C) and a final elongation step at 72°C for 10 min. DNA sequencing was carried out by Macrogen Inc. (Seoul, Korea).

### Real-time PCR

Real-time PCR reactions were performed in the RotorGene Q PCR System (Qiagen) using the KAPA SYBR FAST qPCR Kit (Kapa Biosystems). The 20 µl PCR reactions contained 2 µl of a 1∶100 diluted cDNA sample; 200 nM of each primer and 1× KAPA Master Mix. The cycling protocol was as follows: initial denaturation for 10 min at 95°C followed by 40 cycles of 3 s at 95°C, 20 s at 60°C; 1 s at 72°C. Fluorescence was measured after the extension phase at 72°C. The PCR products were subjected to a melting curve analysis, that commenced at 52°C and increased at 0.5°C s-1 up to 95°C, with a continuous fluorescent measurement. Specific amplification was confirmed by a single peak in the melting curve. For each experimental condition stationary phase genomic DNA was extracted from two independent cultures. The reactions for each target gene were performed in triplicate and in the same PCR run. DNA 10-fold dilutions (ranging from 10 ng to 1 pg) of corresponding PCR amplicons were used to generate a 5-point standard curve for every gene by using the Cycle Threshold (Ct) value versus the logarithm of each dilution factor. Reaction efficiency (E = (10(−1/slope))−1) for every gene was derived from the slope of the corresponding standard curves. Amplicon quantities were calculated from the standard curve by the software Rotor Gene Q Series Software 2.0.2 (Qiagen) set with default parameters. Each experiment included a no template control.

### Mitomycin C and UV treatment

To produce DNA damage, 1 L mid exponential phase cells (3 days old) were concentrated 100 fold and treated with 1 µg/mL mitomycin C (CalBiochem) or exposed to UV-C (3 min at 200 J/m2, 254 nm) for 5 min and then allowed to recover for two generation times (16 h) in fresh media at 40°C and 200 rpm. Total genomic DNA was extracted as described above. The *attL* and *attR* recombination sites flanking the ICE and the chromosomal attachment site (*attB*) were PCR amplified using 10 ng of total genomic DNA and the primers listed in **Table S7**. All experiments were performed in triplicate.

## Supporting Information

Table S1 *A. caldus* strain-specifc gene lists.
(XLSX)

Table S2 Taxonomic origin of *A. caldus* ATCC 51756 and *A. caldus* SM-1 exclusive genes.
(XLSX)

Table S3 Megaplasmid mpAca_TY_.1 and plasmids pAca_TY_.1 and pÀca_TY_.2 annotation and gene module information.
(XLSX)

Table S4 Predicted IS families and features associated to IS-rich regions in the genome of *A. caldus* ATCC 51756 and *A. caldus* SM-1.
(XLSX)

Table S5 ICE and IME Trb gene cluster synteny analysis.
(XLSX)

Table S6 *A. caldus* strains used in this study.
(XLSX)

Table S7 Oligonucleotide primers used in this study.
(XLSX)
