# Correction: Architecture and Gene Repertoire of the Flexible Genome of the Extreme Acidophile *Acidithiobacillus caldus*

**DOI:** 10.1371/journal.pone.0122682

**Published:** 2015-03-13

**Authors:** The PLOS ONE Staff

The grant number 3130441 is missing from the funding section. The correct funding statement is.

This work was supported by the Fondo Nacional de Desarrollo Científico y Tecnológico Fondecyt (http://www.conicyt.cl/fondecyt/) [grant numbers 1100887, 1090451, 3130441], and the Programa de Financiamiento Basal de la Comisión Nacional de Investigación Científica y Tecnológica (http://www.conicyt.cl/pia/category/linea?s-del-programa/creacion-consolidacion-ce?ntros/ [grant number PFB16]. The funders had no role in study design, data collection and analysis, decision to publish, or preparation of the manuscript.
